# Invasive ductal breast cancer with focal mucinous differentiation in a 38-year-old woman: Case report and literature review

**DOI:** 10.1097/MD.0000000000042465

**Published:** 2025-05-30

**Authors:** Xiaolong Ma, Wenjing Shi, Ningning Ren, Yawei Zhang, Xingsong Tian

**Affiliations:** a Department of Breast and Thyroid Surgery, Shandong Provincial Hospital Affiliated to Shandong First Medical University, Jinan, Shandong, China; b Department of Breast Surgery, The Second Affiliated Hospital of Zhengzhou University, Zhengzhou, Henan, China; c Cheeloo College of Medicine, Shandong University, Jinan, Shandong, China.

**Keywords:** case report, focal mucinous differentiation, invasive ductal breast cancer

## Abstract

**Rationale:**

Breast cancer (BC) is a common malignant tumor with a poor prognosis that requires early treatment. Invasive ductal BC with focal mucinous differentiation in a 38-year-old woman is rarely reported.

**Patient concerns:**

A 38-year-old woman presented to our clinic 3 months after discovering a breast mass that had increased in size during this period. Enhanced computed tomography showed an irregular mass with lobulated edges and burrs at the 9 o’clock position on the patient’s right breast. An early enhanced scan showed significant heterogeneous enhancement with continuous enhancement.

**Diagnoses:**

The patient’s postoperative pathology suggested that the patient had an infiltrating ductal BC with focal mucinous differentiation.

**Interventions:**

The patient underwent right breast mass resection first. After the pathological diagnosis was confirmed, the patient underwent right breast resection and right axillary lymph node dissection.

**Outcomes:**

The patient’s postoperative recovery was good. To date, the patient has not experienced any symptoms of recurrence.

**Lessons:**

Owing to the poor prognosis of BC, patients with abnormal breast masses should be diagnosed and treated as soon as possible. Further prognosis research on the invasive ductal carcinoma with focal mucinous differentiation occurring in young individuals is warranted.

## 1. Introduction

Breast cancer (BC) is a common malignant tumor among women. The incidence rate varies among races, approximately 99.2 to 133.7 per 100,000 person-years.^[1]^ Its mortality rate is approximately 11.7 to 27.6 per 100,000 person-years, ranking 2nd after lung cancer among malignant tumors.^[1]^ On the basis of pathology, BC is divided into 3 types: noninvasive, invasive nonspecific, and invasive specific. Noninvasive cancers include ductal carcinoma, in situ lobular carcinoma, and papillary eczema-like carcinoma. Noninvasive cancers occur at an early stage and has a good prognosis. Invasive nonspecific carcinomas are the most common type of BC and include invasive ductal carcinoma (IDC), invasive lobular carcinoma, and adenocarcinoma. Invasive special cancers include papillary carcinoma, mucinous carcinoma (MC), and squamous cell carcinoma and have a relatively low incidence. In the World Health Organization Classification of Breast Tumours (5th edition), based on the proportion of mucinous components, MC is categorized into 2 types: pure MC and mixed MC.^[2]^ In our previous clinical practice, we found that BC often involves mixed cancers, and the most typical mixed type is that of breast IDC with intraductal carcinoma. Here, we report a case of invasive ductal BC with focal mucinous differentiation in a 38-year-old woman, a rare occurrence in clinical practice. We hope to provide a reference for diagnosing and treating invasive ductal BC with focal mucinous differentiation occurring in young individuals.

## 2. Case presentation

### 2.1. Patient information

**Figure 1.**
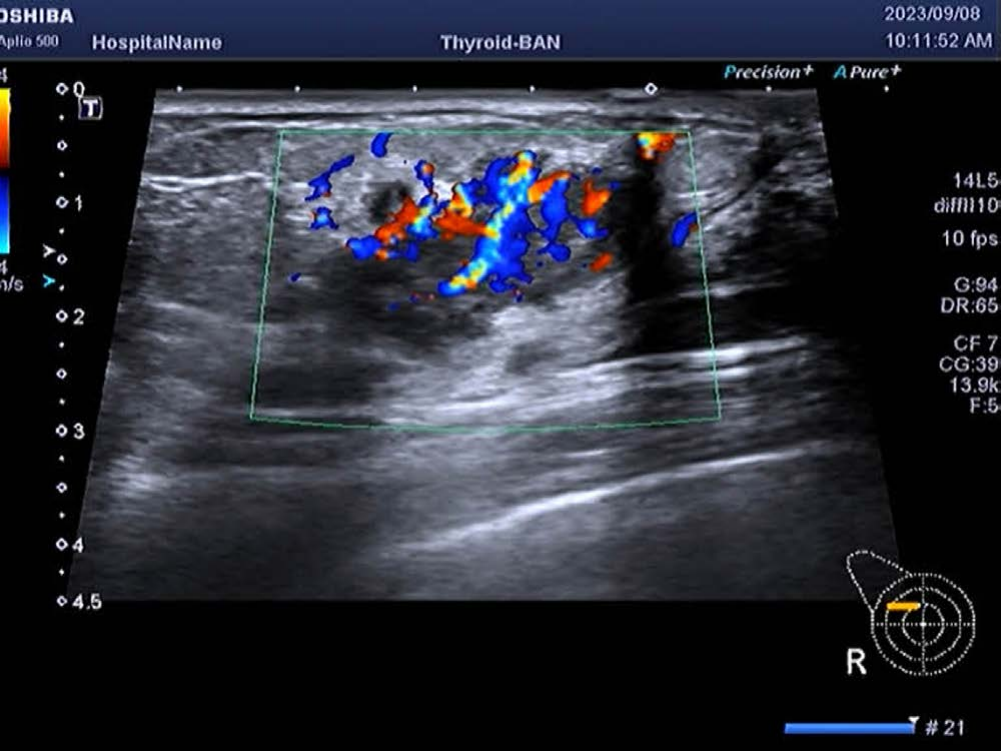
The breast ultrasound of the patient indicated that there was an abnormal nodule in the right breast, which was rich in blood flow signals, and breast cancer was more likely.

The patient was a 38-year-old female who noticed a nodule on the right breast, approximately the size of a mung bean, without any accompanying symptoms. However, she presented to the hospital for treatment 3 months later, when the nodule size had increased. The patient had no other diseases and denied any history of tumors or familial genetic diseases. After physical examination, we found that the nodule could be palpated at the 9 o’clock position on the patient’s right breast; it was approximately 3.5 × 2.0 × 3.0 cm in size, hard in texture, with unclear boundaries and poor mobility. We also found another hard lymph node that could be palpated in the right armpit, approximately 1.5 × 1.0 cm in size, hard in texture, and with poor mobility. No obvious nodules were palpable on the left breast and armpits. Considering the possibility of BC, ultrasound examination of the breast and axillary lymph nodes (ALNs) was recommended. Breast ultrasound results showed (Fig. 1) a hypoechoic nodule with a size of approximately 3.2 × 1.7 × 2.7 cm in the right breast gland, which was irregularly shaped with rough edges and low echoes and had thick blood flow signals detected internally. Multiple lymph node echoes were detected in the right armpit, with the largest being approximately 1.5 × 0.9 cm in size with cortical thickening and an unclear corticomedullary boundary. Notably, both nodules were identified as Breast Imaging Reporting and Data System (BI-RADS) category 5 lesions.

We preliminarily diagnosed the patient likely to have both BC and ALNs. We suggested that the patient undergo core needle biopsy (CNB) to further clarify the nature of the tumor. However, the patient opted for surgical treatment as soon as possible.

### 2.2. History of the current disease

**Figure 2.**
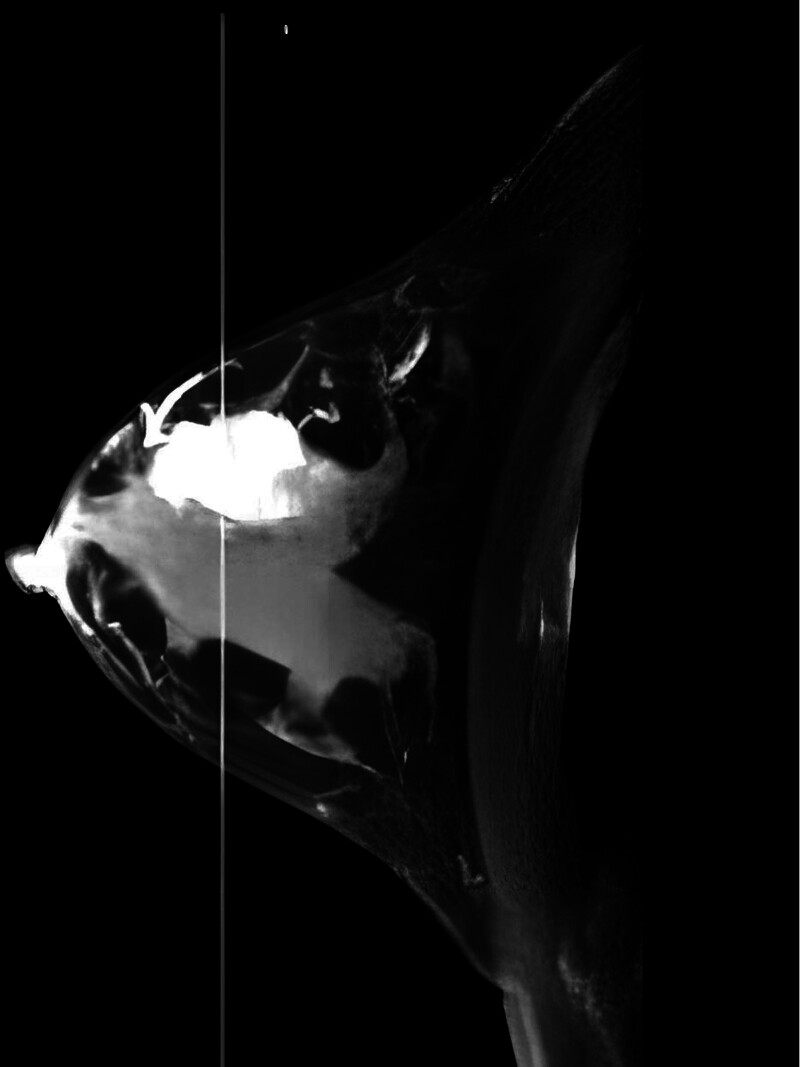
The patient’s breast CT results showed a significantly enhanced mass on the outer side of the right breast. CT = computed tomography.

After the patient was admitted to the hospital, a thorough physical examination was conducted, and breast-enhanced computed tomography (CT) was performed. The findings indicated an irregular mass (approximately 2.5 × 2.3 cm in size) at the 9 o’clock position in the right breast (Fig. 2) with lobulated edges and burrs. Obvious heterogeneous enhancement could be observed at the early stage of enhanced scanning, consistent with the CT findings of BC, and the BI-RADS classification was 5. Notably, other physical examinations revealed no apparent abnormalities. The patient’s breast tumor markers carcinoembryonic antigen, carbohydrate antigen 125, carbohydrate antigen 153, and carbohydrate antigen 199 were all within normal ranges.

### 2.3. Diagnostic assessment

Through a series of examinations, the patient was initially diagnosed with right BC with ALN metastasis; the tumor stage was cT2N1M0 or IIB. The patient’s condition met the surgical indications, with no surgical contraindications. Therefore, surgical treatment was initiated as soon as possible.

### 2.4. Therapeutic intervention

**Figure 3.**
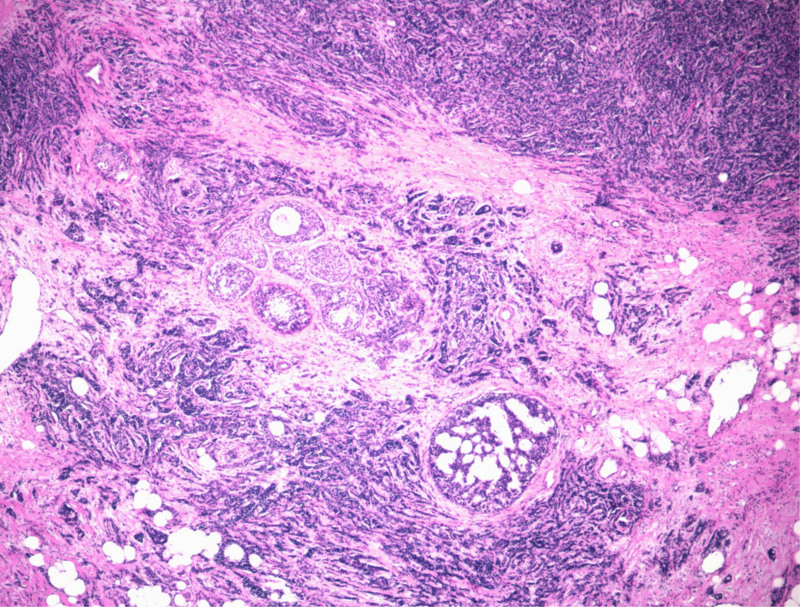
The postoperative paraffin pathological results of the patient suggested invasive ductal carcinoma mixed with mucinous carcinoma of the breast, and macrometastasis in the axillary lymph nodes.

**Figure 4.**
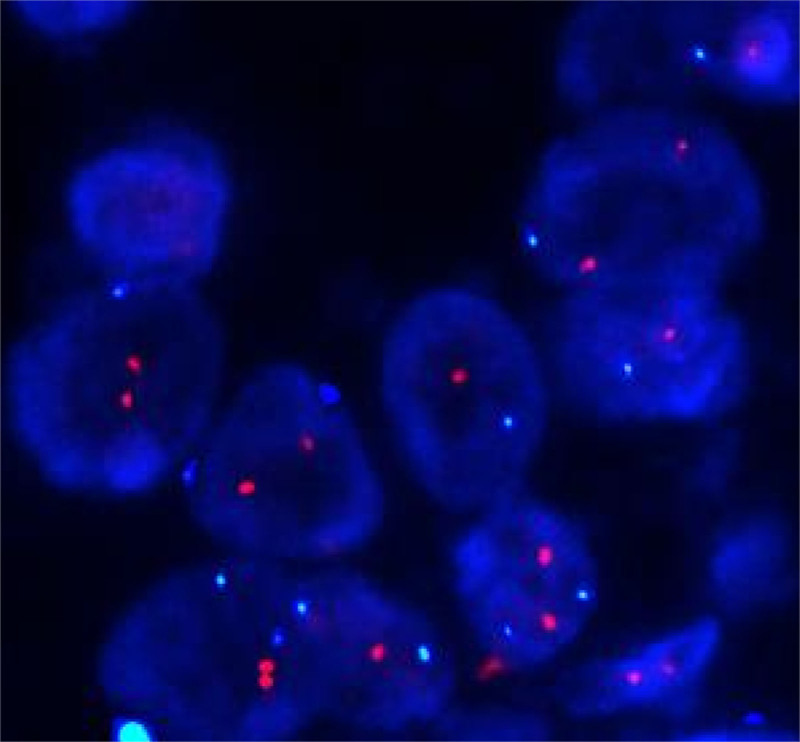
The patient’s FISH test results indicated that Her-2 is negative. FISH = fluorescence in situ hybridization, Her-2 = human epidermal growth factor receptor 2.

We thoroughly explained the differences and risks of various surgical methods to the patient, and she decided to undergo the right breast mass resection first. After resection of the right breast mass, the patient’s intraoperative pathology indicated invasive carcinoma of the right breast. Therefore, right breast resection was performed, followed by right ALN dissection. In the patient’s right armpit, we found multiple swollen lymph nodes with a hard texture and a high probability of metastasis. This possibility was discussed with the patient and her family members before surgery. The patient and her family members refused sentinel lymph node biopsy and requested a direct right ALN dissection. Postoperative pathological indication of the patient was as follows: the right breast showed IDC, 5% of which was MC, with the area of 3.5 × 2 cm; 1/12 of the right ALNs showed metastatic cancer, indicating macroscopic metastasis (Fig. 3). The immunohistochemical results were as follows: estrogen receptor+ (80%), progesterone receptor+ (70%), androgen receptor+ (90%), human epidermal growth factor receptor 2 (Her-2) (2+), Ki-67 (90%), E-Cad (+), P120 (+), P63 (−), CK5/6 (−). After surgery, the patient underwent fluorescence in situ hybridization testing, and the results indicated a negative Her-2 (Fig. 4).

### 2.5. Follow-up and outcomes

**Figure 5.**
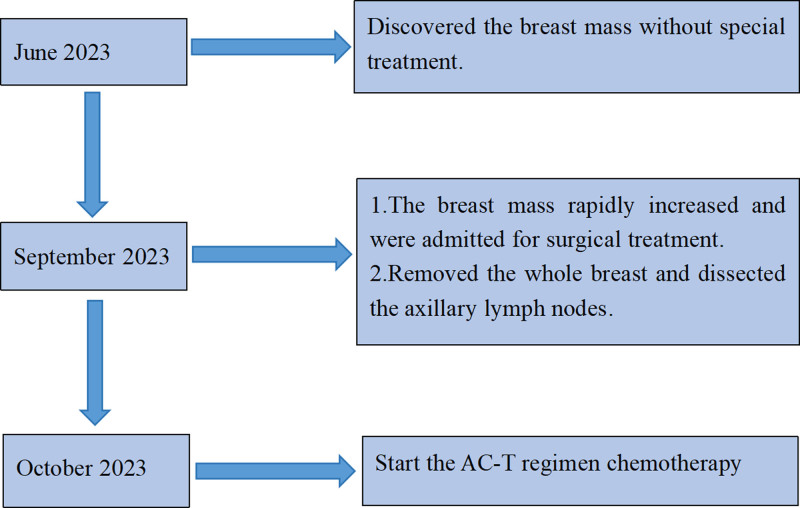
Timeline. AC-T = adriamycin and cyclophosphamide to taxol.

The patient recovered well after surgery and was advised to rest to prevent infection. After the surgery, the patient visited the oncology department for treatment and began chemotherapy with 8 cycles of the adriamycin and cyclophosphamide to the taxol regimen. To date, the patient has not experienced any symptoms of recurrence. A timeline of the patient’s diagnosis and treatment process is shown in Figure 5.

## 3. Discussion

BC is one of the most common malignant tumors, and its incidence and mortality rates differ by age, race, and country. The total incidence rate of this disease increases annually by 0.9%.^[1]^ BC tends to occur in individuals aged >50 years, and its mortality rate is also high in this age range. The highest incidence rate of BC is found in Caucasians (approximately 133.7 per 100,000 person-years), and its mortality rate is highest in Africans, approximately 27.6 per 100,000 person-years, 40% higher than that in Caucasians.^[1]^ Over half of the patients with BC were still in the early stage when they were diagnosed, and approximately 4% of them already had metastasis in other parts at diagnosis.^[3]^ Therefore, timely BC diagnosis and treatment are very critical. Invasive breast cancer (IBC) is the most common type of BC, accounting for >80% of cases.^[4]^ IBC cells invade the basal membrane of the breast ducts or lobules and the stroma. Compared with noninvasive BC, IBC has a relatively poor prognosis, with a mortality rate of 3%.^[1]^ MC is a rare type of IBC that accounts for approximately 3% and 4% of all BC and IBC cases, respectively.^[5]^ Its typical feature is the presence of many extracellular mucins, and its prognosis is better than that of other types of BC. According to the postoperative pathological results, the patient’s diagnosis is considered IDC with focal mucinous differentiation. While not meeting World Health Organization criteria for mixed MC, this case represents an unusual presentation of coexisting histologic patterns, especially among relatively young women.

Owing to the high degree of BC malignancy, accurate and early diagnosis before surgery is necessary to provide treatment as soon as possible. According to the National Comprehensive Cancer Network (NCCN) guidelines, ultrasound and radiography examinations should be performed before surgery for patients with nonmetastatic IBC. CNB should be performed, if necessary, to further clarify the diagnosis.^[6]^ In breast ultrasound, images of BC often show irregular hypoechoic masses with unclear boundaries, lobulated and burred, with an aspect ratio > 1, and rich blood flow signals. On enhanced CT/magnetic resonance, malignant breast masses were observed as significantly bright areas, indicating significant enhancement. This observation suggests abundant blood vessels and sufficient blood flow in the mass, indicating that the mass is likely to be a malignant tumor. Currently, regarding imaging examination, breast masses are commonly classified using the BI-RADS. According to the BI-RADS principle and based on the imaging manifestations of breast masses, they can be divided into 6 levels: 1, 2, 3, 4 (4A, 4B, 4C), 5, and 6.^[7]^ Grade 4 or higher indicates that the nodule may be malignant. With an increase in grade, the probability of malignancy gradually increases, whereas grade 6 refers to BC pathologically diagnosed. On the basis of the BI-RADS principle, a preliminary judgment regarding benign and malignant breast masses can be made without histological examination.

The 2 methods used for surgical treatment of the affected breast include breast-conserving surgery (BCS) + radiation therapy and whole-breast resection (WBR). BCS involves removing the tumor and the surrounding normal breast tissue (to ensure a safe margin distance), preserving most of the breast tissue, and assisting in postoperative radiotherapy. WBR involves removal of all breast glands and tissues, such as nipples and areolas. A study including 18,997 patients with BC showed that the total survival period of patients who underwent BCS + radiotherapy was better than that of those who underwent WBR (relative risk: 0.64, 95% confidence interval: 0.55–0.74).^[8]^ However, another study including 3536 patients showed no significant difference in prognosis between the 2 groups (hazard ratio: 0.81, 95% confidence interval: 0.64–1.03).^[9]^ Therefore, further research is required to determine the best treatment method for patients with BC. For patients with positive ALNs, CNB or sentinel lymph node biopsy can be performed to further evaluate the armpit’s condition. However, in the present study, the patient refused both procedures. It is understandable and acceptable to perform ALN dissection directly when the likelihood of ALN metastasis is high; however, ALN dissection may be unnecessary for some patients.

After surgery, a decision should be made on whether to assist with radiotherapy or chemotherapy based on the postoperative pathological results. According to the postoperative pathological results, the patient’s molecular typing was found to be estrogen receptor and progesterone receptor positive and Her-2 negative, with ALN macrometastasis. Postoperative endocrine therapy and neoadjuvant chemotherapy are necessary for IBC. According to the NCCN guidelines, the preferred chemotherapy regimen is the adriamycin and cyclophosphamide to taxol regimen, which involves first administering a 4-cycle adriamycin + cyclophosphamide therapy and then switching to taxol-based drugs for the next 4 cycles;^[6]^ our patient is currently undergoing this treatment. Postoperative adjuvant endocrine therapy can be considered for histological types with good prognosis, such as MC,^[10]^ whereas chemotherapy is unnecessary.

## 4. Conclusion

BC is a common malignant tumor with a relatively high degree of malignancy and poor prognosis. When a patient is suspected of having BC, imaging examinations (such as ultrasound and radiography) should be performed as soon as possible. If necessary, CNB can be performed to ensure a clear diagnosis. After diagnosis, the patient should undergo immediate surgical treatment. IDC is often observed in clinical practice, but IDC with focal mucinous differentiation is rarely seen, especially among relatively young women. The NCCN guidelines suggest different treatment options for IDC and MC. Currently, there are no specific or definitive suggestions for IDC with focal mucinous differentiation, and hence, further research in this direction is required. The patient and her family members are satisfied with our treatment, and she has been persisting in follow-up examinations after surgery. Wishing her good health.

## Author contributions

**Writing – review & editing:** Xiaolong Ma, Wenjing Shi.

**Resources:** Ningning Ren.

**Formal analysis:** Yawei Zhang.

**Supervision:** Xingsong Tian.
